# A Perspective on the (Rise and Fall of) Protein β-Turns

**DOI:** 10.3390/ijms232012314

**Published:** 2022-10-14

**Authors:** Alexandre G. de Brevern

**Affiliations:** Université Paris Cité and Université des Antilles and Université de la Réunion, INSERM UMR_S 1134, BIGR, DSIMB Team, F-75014 Paris, France; alexandre.debrevern@univ-paris-diderot.fr; Tel.: +33-1-44493000

**Keywords:** secondary structure, sequence structure relationship, secondary structure assignment method, DSSP, bend, hydrogen bonds, AlphaFold 2

## Abstract

The β-turn is the third defined secondary structure after the α-helix and the β-sheet. The β-turns were described more than 50 years ago and account for more than 20% of protein residues. Nonetheless, they are often overlooked or even misunderstood. This poor knowledge of these local protein conformations is due to various factors, causes that I discuss here. For example, confusion still exists about the assignment of these local protein structures, their overlaps with other structures, the potential absence of a stabilizing hydrogen bond, the numerous types of β-turns and the software’s difficulty in assigning or visualizing them. I also propose some ideas to potentially/partially remedy this and present why β-turns can still be helpful, even in the AlphaFold 2 era.

## 1. Forewords

This short pedagogical review comes from many discussions about β-turns that I have had with students and specialists in the field. As I have been surprised too many times, I decided to document my reflections on these unfortunate misunderstandings. This review is directed toward the younger generation, but it could be helpful to more advanced researchers. Music can be context-specific but is also beneficial in multiple pathologies [1,2]; you will find a specific song associated with each section. The content of the song is correlated with the content of the paragraph (see Supplementary List S1). Of course, one can read the review without listening to it directly.

This article is dedicated to Prof. N. Srinivasan, a true master of protein structural bioinformatics, who passed away on 3 September 2021 [3,4,5,6].

## 2. The Alpha and the Omega of the β

“*I see a red door and I want it painted black, No colours anymore, I want them to turn black*” (Rolling Stones, Paint It Black [7]). This song, accompanied by Brian Jones’ sitar, was released two years before the publication of a seminal work by C.M. Venkatachalam. In his 1968 paper, Venkatachalam underlined with strong arguments the existence of the third most occurring secondary structure, i.e., the β-turn [8]. While the α-helix and the β-sheet roughly represented 1/3rd and 1/5th of the amino acids, the β-turn was only slightly less frequent than β-sheet [9,10]. These characteristics remained true whatever the used secondary structure assignment approaches [11]. Thus, in a 3D protein structure analyzed with the α-helices, the β-sheets and the β-turns, less than 1/5th of the amino acids remained associated with the coil state [11]. For a globular structure to form, the polypeptide must fold back onto itself. This was provided by the reverse turn (i.e., the β-turn). Thus, it should be a prevalent structure. β-turns were often referred to as aperiodic or non-regular, while α-helices and β-sheets were the regular and repetitive secondary structure states.

The definition of β-turns was quite simple and corresponded to a quick return of the protein backbone. One β-turn was composed of four consecutive residues, while the γ-, α- and π-turns were composed of three, five and six residues, respectively [10,12,13,14,15,16]. This quick return created a proximity between residue *i* and *i* + 3, mainly described by a distance of less than 7.0 Å (or 7.5 Å) between Cα*_i_* and Cα*_i+3_*. This value was compatible with the classical hydrogen bond of the protein backbone, as observed in repetitive secondary structures [17,18].

However, since I published nearly 6 years ago a study on β-turns [19], I have had a number of questions and remarks about β-turn frequencies and properties.

In the following sections, I discuss the issues of β-turn assignment rules (see Section 2.1), multiple overlapping of turns (see Section 2.2), differences between turns and bends (see Section 2.3), β-turn types (see Section 2.4), dedicated software able to assign β-turn types (see Section 2.5), visualization (see Section 2.6), confusion with other local protein conformations (see Section 2.7) and new classifications (see Section 2.8). The ubiquitin-conjugating enzyme protein structure from *Arabidopsis thaliana* (PDB id 2aak [20]) will be used as an illustration. The protein structure assignment will be made with well-known DSSP [21] and (DSSP-related) PROMOTIF software [22].

### 2.1. Superimposition of the β-Turns with Other Regular Structures

**Figure 1:**
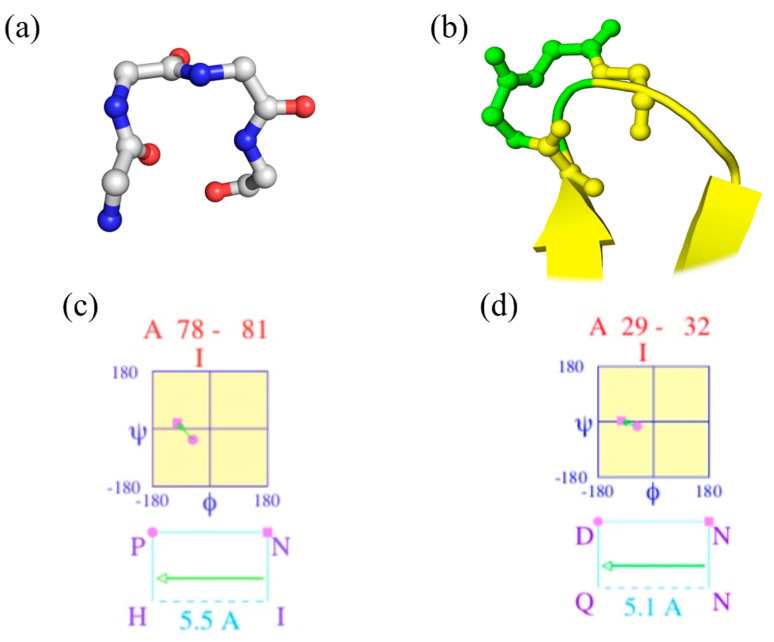
*β-turn visualization of ubiquitin-conjugating enzyme*. (**a**,**c**) Magnification on positions 78–81 and (**b**,**d**) 29–32 of ubiquitin-conjugating enzyme (PDB id 2aak [20]), with (**a**,**b**) 3D visualization with PyMOL [24], and (**c**,**d**) Ramachandran map obtained by PROMOTIF software [25].

*“Walk in silence, Don’t turn away, in silence; Your confusion, My illusion”* (Joy Division, Atmosphere [23]). Figure 1a shows a beautiful classical β-turn (position 78–81 of PDB id 2aak [20]), while Figure 1c provides the corresponding Ramachandran plot of two central residues generated by PROMOTIF. It is a perfect β-turn; this example shows that seeing helices, strands, loops (coil) and turns is a simple exercise.

**Figure 2:**
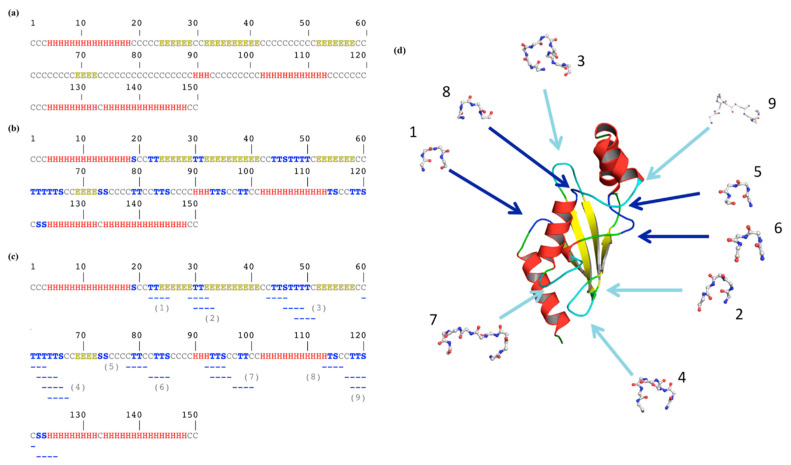
*β-turn localization on ubiquitin-conjugating enzyme*. Secondary structure assignment is performed with (**a**) 3-state DSSP [21], (**b**) 8-state DSSP [21] and (**c**) with PROMOTIF [25], and turns are added and numbered; (**d**) these latter are located on the ubiquitin-conjugating enzyme protein structure using PyMOL [24]. Helices are shown in red, strands in yellow, coil in green and turns in blue when they are unique and cyan when they are overlapping. Numbers are similar between (**c**) and (**d**).

Figure 2 underlines a more complex story. Figure 2a shows the classical three-state assignment for this protein with the highly famous DSSP [21]. With this three-state assignment, the loops (coil) are in the majority (letter “C”, occurrence of 46%), followed by the α-helices (“H”, 36%) and finally the β-sheets (“E”, 18%).

Figure 2b is more complex to analyze as β-turns were added. DSSP assigned β-turns, with two different letters (“T” for the turn and “S” for the bend; see Section 2.3). The coil state decreases by more than half; the residues exclusively loops being only 21.3%, the turns represent 24.6% of the residues (with 17.3% of “T” and 7.3% of “S”). Thus, the β-turn is the most frequent secondary structure in this protein.

One point that could surprise the non-specialist is that these β-turns, composed of four consecutive residues, are not represented by a succession of four equivalent letters. For example, residues 22 and 23 are a β-turn (“T”) immediately before a β-strand (positions 24 to 29). The following β-turn (positions 30 and 31) is surrounded by these two β−strands, forming an anti-parallel β-sheet. Indeed, β-turns overlap helical and extended regions.

In fact, after the extremity distance criteria, the second assignment rule is that (i) the central residues of the β-turns must be non-helical, and (ii) in the case of β-strands, at least one residue must be associated with a coil state. Thus, one β-turn is not necessarily composed of four consecutive coil residues.

Figure 1b focuses on another β-turn of the same protein (positions 29 to 32) that overlaps β-strands, while Figure 1d provides the Ramachandran plot. As DSSP does not directly provide the beginning and last residue of each β-turn, another tool must be used, namely PROMOTIF [22]. For this protein, 18 β-turns are assigned by PROMOTIF (see Figure 2c), and 1/3 overlap with α-helix or β-strand residues. Please note that PROMOTIF is very similar to DSSP assignment as they have a consensus of 95% for the assignment of α-helix, β-sheet and coil [26,27,28,29].

Hence, it is imperative to note that β-turns are not composed of only residues that could be considered as loops/coil residues. This underlines the first difficulty.

### 2.2. Superimposition of the Assignment with Other β-Turns

“*You spin me right ‘round, baby, Right ‘round like a record, baby*” (Dead or Alive, You Spin Me Round (Like a Record) [30]). The vision of a β-turn as a reversal in the polypeptide chain direction led to the ideal β-turn shown in Figure 1a. As presented in the previous section, it is not as simple. 

Moreover, a second inconvenience is that, in addition to the potential overlapping of α-helices and β-sheets, the β-turns may overlap with other β-turns. Early systematic studies assessed that more than half of the β-turns are associated with multiple β-turns [31,32].

In our example, only the β-turn found at positions 21–24 (noted 1 in Figure 2c) is alone, with no other β-turn in the neighborhood. The 17 remaining β-turns are overlapping by one, two or three residues (n°2, n°3, n°4, n°7 and n° 9) with another one; some are independent but adjacent to another one (n°5 and n°6 within n°7, n°8 and n°9), while certain ones are with overlapping residues and successive β-turns, i.e., n°7. This shows that β-turns have a much more complex environment than simply loops.

Thus, it is essential to notice that β-turns are not often independent. This provides the second difficulty for their analyses and usages.

### 2.3. With or without Hydrogen Bonds

“*With or without you, Through the storm we reach the shore*” (U2, With Or Without You [33]). The number of Secondary Structure Assignment Methods (SSAM) developed is high. At least 40 different ones can be found in the literature [29,34], e.g., DEFINE [35], SEGNO [36], XTLSSTR [37], P-SEA [38], KAKSI [39], DLFSA [40] or P-CURVE [41]. They are based on very different metrics and rules, leading to a consensus of only 80% [26,28]. Only a limited number of SSAMs assign β-turns, and this often differs as the assignment of the α-helix and β-sheet is also different.

DSSP [21] (and two of the derived approaches, namely SECSTR [42] and PROMOTIF [22]) has the particularity to assign two classes of β-turns, i.e., turns (or β-turns with stabilizing hydrogen bonds, associated with the letter “T”) and bends (or β-turns without stabilizing hydrogen bonds, associated with the letter “S”). STRIDE [43] (the second most used SSAM) and most of the analyses on β-turns aggregate both to the turn secondary structure. 

This seems to be a very minor issue as these two states are highly similar, as (i) the repetitive secondary structure rules are identical and (2) the Cα–Cα distance threshold is always true when a hydrogen bond is present. In some papers, it is even considered as a unique state, e.g., “The beta-turn, which has also been referred to as the beta-bend, beta-loop or reverse turn” in [44]; they are always merged for prediction purposes [45,46].

**Figure 3:**
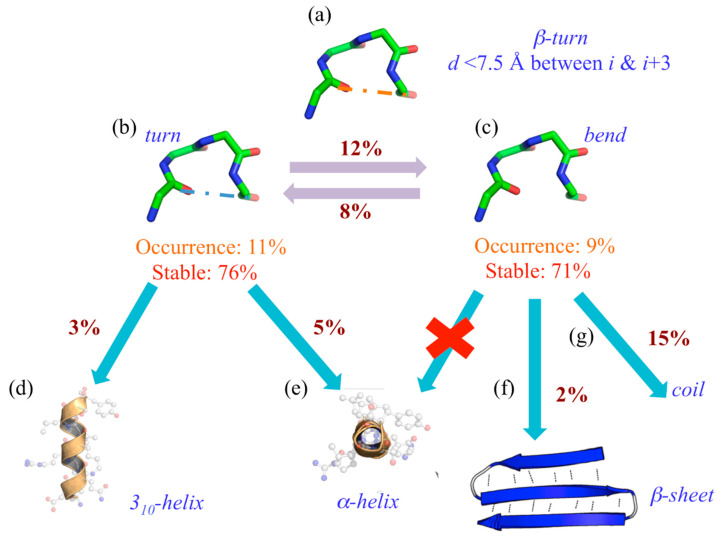
*Turns and bends are not going in the same direction*. (**a**) The general definition of β-turns; they are assigned as (**b**) turn (“T”) and (**c**) bend (“S”) by DSSP, and their occurrences and stability rates during MDs are provided. When they do not stay associated with their original assignment, they can be found assigned to (**d**) 3_10_-helix, (**e**) α-helix, (**f**) β-sheet and (**g**) coil state.

Nonetheless, it is not as simple as expected. Indeed, using 169 protein structures submitted to molecular dynamics, we have observed a drastic difference in the turn’s dynamics depending on its stabilizing hydrogen bond or its absence (see Figure 3) [47]. 

Indeed, its assignment can be considered extremely simple (see Figure 3a). When 20% of the residues are assigned to β-turns, 11% are turns (“T”) with a hydrogen bond (see Figure 3b) and 9% without, namely bends (“S”). These two states can be considered well stable (and equivalently so), with 76% of “T”s and 71% of “S”s never changing state during the dynamics. Interestingly, 12% of “T” goes to “S” and 8% does the reverse. The most interesting point is that when they actually change state, “T” goes at 5% to the α-helix (see Figure 3e) and at 3% to the 3_10_-helix (see Figure 3e), “S” never goes to helical structures (see Figure 3e) at 15% to coil state (see Figure 3g) and quite unexpectedly 2% to β-sheet (see Figure 3f).

Hereafter, it is interesting to notice that β-turns with or without hydrogen bonds are not entirely equivalent. This is the third difficulty for their analyses and usages.

### 2.4. The Mess for Types (and Some Types Are for Nothing)

*“That ain’t workin’ that’s the way you do it, Money for nothin’ and your chicks for free”* (Dire Straits, Money for Nothing [48]). In his seminal work, C.M. Venkatachalam did not only present the β-turns; he also defined six types based on their central (ϕ, ψ) angle values using a threshold of +/- 30° (and one allowed at more than +/−45°). He proposed types I, II and III, with their reverse versions of types I’, II’ and III’, and a last type named type IV (for all β-turns that cannot be associated with one of the first six) [8]. 

As very well presented by J. Richardson in her masterpiece, “The anatomy and taxonomy of protein structure” [18], the evolution of β-turns had not followed a simple pathway. To summarize, (i) type III was erased due to confusion with 3_10_-helix (indeed, 90% of the former type III can assigned to 3_10_-helix [19]; type III’ disappeared too, being close to type I’) [19,49]; (ii) new types V and VII were added [9] and erased [18]; (iii) type VI was created to take into account the presence of *cis*-Pro at position *i* + 2 (three different types VI exist) [9,18]; and (iv) C.M. Wilmot and J. Thornton defined type VIII [50] and it is maintained as it is highly occurring.

The current canonical classification considers nine types (I, I’, II, II’, IV, VI_a1_, VI_a2_, VI_b_ and VIII), as performed by PROMOTIF. However, except for type I (38% of β-turns), the most frequent β-turn is type IV (32%), the β-turn defined as not associated with a specific β-turn definition, and some β-turns’ occurrence is low (VI_a2_ 0.2%, VI_a1_ 0.7%, VI_b_ 0.9%, II’ 2.5% and I’ 4% [16]). This typology is so not as easy as 3_10_-, α- and π-helix, for instance. Indeed, this classification is slightly higher than the number of states assigned by DSSP (only eight secondary structure states).

This adds a further degree of complexity to the use of the β-turn.

### 2.5. Only One Ancient Tool to Assign

*“In the year 2525, If man is still alive, If woman can survive, They may find.*” (Zager & Evans, In the Year 2525 (Exordium & Terminus) [51]). A general issue in informatics is the obsolescence and disappearance of scientific tools [52], and it is particularly true in bioinformatics and computational biology [52]. Nearly all the SSAMs are currently unavailable or not usable (e.g., compiled versions for obsolete systems or unavailable dependencies or compiler versions). 

DSSP [21] was published nearly 40 years ago and is still operational today. It is one of the rare cases of continually updated software provided by different scientific teams (even with the changes of informatics languages, but happily retaining the same output) [53]. 

In this field, young scientists may discover that the approach that is considered to be the state of the art for turn assignment, namely PROMOTIF [22], was published in 1996. It is also slightly complex for the non-specialist to use as it is based on an old Fortran version and could need some code modifications to work (Old Fortran has been forever linked to Futurama’s Bender, since the first episode entitled *Space Pilot 3000* [54]).

**Figure 4:**
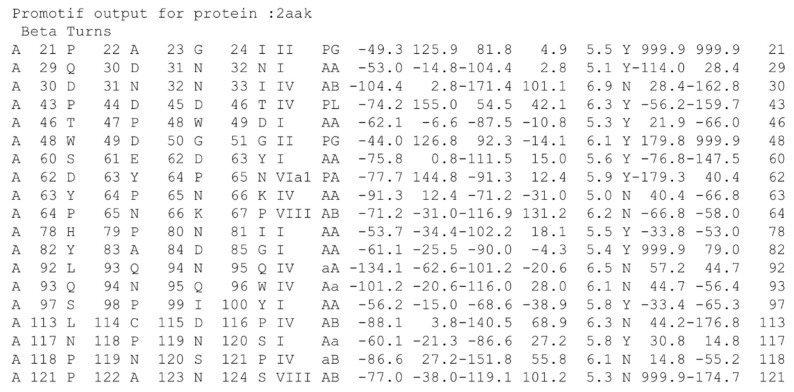
*PROMOTIF output for β-turn assignment of ubiquitin-conjugating enzyme*. I provide the chain letter, and the positions and typed of 4 residues implicated in the β-turn, with its type, corresponding Ramachandran region, the central ϕ and ψ angle values, the distance between the first and last Cα, the presence (or not) of a hydrogen bond and the χ angle values of central residues.

As an old specialist, I am impressed with its operation and output (see Figure 4 for an example), but a more recent and well-designed software could be needed, and probably also a more efficient output. In a few words, students do not like to use it. 

Hence, it is important to notice that β-turn assignment with the most specialized tool could be an issue. This is an additional difficulty and, shortly perhaps, a real problem with the evolution of operating systems and languages.

### 2.6. No (Easy) Specific Visualisation

*“It don’t come easy, You know it don’t come easy”* (Ringo Starr, It Don’t Come Easy [55]). Molecular visualization is fundamental in the current scientific literature, textbooks and dissemination materials; it expands the understanding of biomolecular function [56,57]. Multiple visualization tools perform three-state DSSP-like or STRIDE-like assignment and rendering. Nevertheless, the most used visualization tools, e.g., PyMOL [24] or Chimera [58], do not visually assign β-turns.

**Figure 5:**
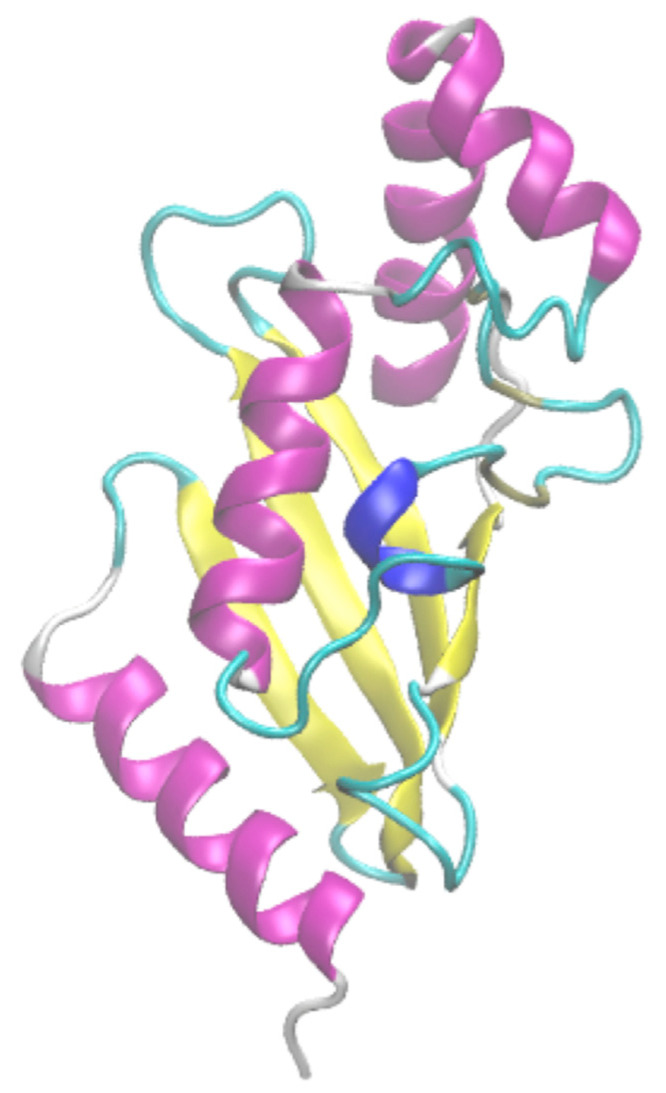
*VMD visualization*. The ubiquitin-conjugating enzyme is represented in an illustration with VMD software [59]. α-helices are in purple, β-sheets in yellow, coil in white, 3_10_ helix in blue and β-turn in cyan.

VMD [59] proposed a simple coloration (see Figure 5). This simple coloration is difficult to follow, as can be seen in Figure 2. Even if different colors were added to define the positions of β-turns, it is not possible to know if (a) the turn is overlapping with regular secondary structures (see Section 2.1), (b) the turn is overlapping with other β-turns (see Section 2.2) or (c) which type it may be (see Section 2.4).

Thus, it is critical to see that β-turn visualization remains an issue and so is a strong limitation. This brings additional complexity to β-turns.

### 2.7. Confusion with Other Local Protein Structures

*“Confusion in her eyes that says it all, She’s lost control, And she’s clinging to the nearest passer by, She’s lost control”* (Joy Division, She’s Lost Control [60]). During the current pandemic, Greek letters have acquired a bad reputation. β-turns are luckily rarely confused with β-sheets. However, they are more associated (i) with γ-turns [61] than expected and (ii) especially with the β-hairpins. The association is logical for both. The first one is simply a shorter turn of three residues (instead of four), while the second is defined as a short connection between consecutive anti-parallel β-strands. The overlapping of γ- and β-turns is not too frequent (γ-turns being six times less frequent than β-turns [14]), and it is more a terminological confusion than a true one. 

β-hairpins, one of the simplest stable protein structural elements, consist of two antiparallel β-sheets joined by a short loop region. They have been found to be very useful for protein β-sheet folding and stability [62,63], especially with some aromatic residues [64]. They are distributed in an impressive number of different folds, such as the β-hairpin repeat proteins [65]. A very large number of β-turns is found in β-hairpins [66], but some are without [67], and a number of β-turns are not found in β-hairpins. It is critical to note that this connecting loop resembles a hairpin and it is logical that β-turns are found inside (as for γ- and α-turns too [66]), but it is not systematic. 

**Figure 6:**
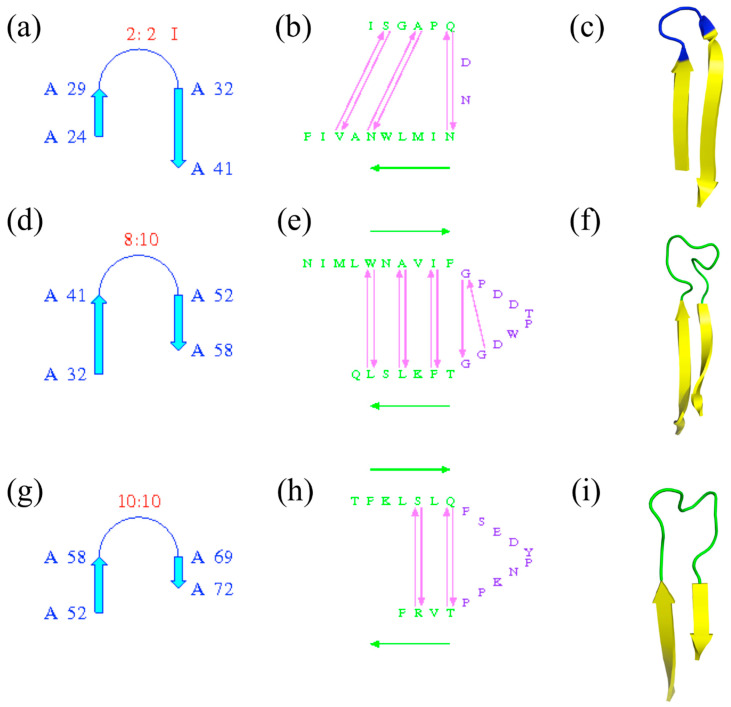
*β-hairpins of ubiquitin-conjugating enzyme*. Three β-hairpins are detected by PROMOTIF software [25], with (**a**–**c**) the first β-hairpin located between β-strands at positions 24–29 and 32–41, (**d**–**f**) the second one between β-strands at positions 32–41 and 52–58 and (**g**–**i**) the third one between β-strands at positions 52–58 and 69–72. (**a**,**d**,**g**) are the schematic representations of β-hairpins with positions and types of β-hairpins, (**b**,**e**,**g**) similar information with associated residues and (**c**,**f**,**i**) PyMOL visualization—in yellow, β-strands; in green, coil; and in blue, the β-turn.

The terms are important and precise; they must be used with care and not lead to confusion: β-turns are often found in β-hairpins, which are formed by the presence of two anti-parallel β-strands, but some β-hairpins are without β-turns and many β-turns are outside the β-hairpins. Figure 6 shows the β-hairpins of ubiquitin-conjugating enzyme; PROMOTIF [25] assigns three β-turns. Of the three, only the first one is associated with a β-turn (of type I; see Figure 6a–c), while the two others are with longer connecting loops without β-turns (see Figure 6d–i).

### 2.8. New Classifications

*“It’s a new dawn, It’s a new day, It’s a new life for me, ooh, And I’m feeling good”* (Nina Simone, Feeling Good [68]). The initial definition of the β-turn by C.M. Venkatachalam [8] remains the most popular one, while the definitions of types have led to nine canonical types [9,10,18,50]. Nonetheless, different studies have provided potential new classifications.

At the beginning of the 1990s, C.M. Wilmot and J. Thornton analyzed the β-turns in the light of Ramachandran plot regions (defined by six regions: αR, βE, βP, αL, γL and ε) for their two central residues [4]. They defined 16 types; half of them have an occurrence of less than 1% [69]. This work can be placed in parallel with concomitant research on secondary super-structures [70] or structural alphabets [71].

Koch and Klebe designed a complex advanced Self-Organizing Map [72,73] to learn every type of turn (from two to six residues in length) [74]. They also discussed the distance between fragment extremities (reaching 10 Å) and ω angle, leading to the description of normal, open and reverse turns. For the β-turn, they thus presented six normal β-turns (five overlapping with classical description [22]), 17 open β-turns (six overlapping with classical description [22]), six of the open β-turns being considered as kink types and finally 18 reverse β-turns. They used this new classification (41 different β-turns) to perform turn prediction with Support Vector Machines [75]. As they were included in Secbase [76], it is nowadays not possible to use them. Following this approach, a new secondary structure assignment method (SSAM) was proposed, namely SCOT, Rethinking the Classification of Secondary Structure Elements [34,77]. This is an entirely new SSAM with the assignment of repetitive structures and β-turns with 7 normal and 33 open types. A Linux CentOS distribution is available.

Using an unsupervised clustering related to Self-Organizing Map [72,73], I proposed an extension of the type IV β-turn, the second most populated β-turn type. Indeed, this type is simply the “*cannot be associated with a defined cluster*” type. Interestingly, four populated clusters were recurrently found, defining four new types, named IV_1_, IV_2_, IV_3_ and IV_4_, allowing the definition of more precisely half of type IV β-turns [19].

R. Dunbrack’s team proposed “A new clustering and nomenclature for β-turns derived from high-resolution protein structures” [78]. They used a density-based clustering algorithm, named DBSCAN [79], and k-medoids, to classify β-turns combining the distance extremities and the three dihedral angles (ϕ, ψ and ω) of central residues. They proposed 18 β-turn types, with 11 new types of β-turn, five of which are sub-types of classical β-turn types. Cross-platform software BetaTurn-Tool18 was proposed to assign them. With the evolution of the package and informatics language, it has become difficult to use it at present. 

Zhang and co-workers introduced a new scheme for classifying β-turns in protein structure description [80]. Each dihedral angle was discretized in an interval of 60°, leading to six possibilities, i.e., for the two central residues, a total combination of 1296 combinations was possible. As seen with a non-redundant dataset of protein structures, already, 583 different types were observed; only the 19 most frequent represented an occurrence higher than 1%. These most frequent clusters were in excellent agreement with classical [22,69], extended [19] and new [78] classifications.

As in many fields, the oldest definitions are often the most used, the best known and therefore the most quoted, sidelining the most recent approaches. A particular point of most of the approaches presented here is their complex analysis (i.e., too many states, which makes them difficult to read) and also, as discussed in Section 2.5, the computational difficulties in using them.

## 3. Conclusions and At Least Some Perspectives

Thus, is it, *“This is the end, Beautiful friend, This is the end, My only friend, the end”*? (The Doors, The End [81]). From these different points presented, it is possible to define four main sets of questions.

The first is probably the least expected. These are purely IT questions (see Section 2.5 and Section 2.6). The current approaches are either (i) not very accurate, e.g., see Figure 2b, where it is difficult to know which four residues are associated with position 19 (a bend), or (ii) difficult to use with PROMOTIF. Similar to others [17,31,46,82], I often make my own codes from DSSP output, reducing the eight states to three, and then applying the distance and containment rules in secondary structures [11,19]. The approach is efficient, but not the most appropriate for reproducible and rigorous scientific research. 

The second one is represented in Figure 1b; the β-turn overlaps with other defined secondary structures. It complicates their utilization. Moreover, they also overlap with themselves, e.g., one β-turn can have its first residue in an α-helix or a β-strand and its last residues are also with another β-turn. Hence, the information could be confused and also no simple visualization can be performed to help the scientist (see Figure 2c,d). 

The third issue is the question of distance extremities. Indeed, it is often believed that all β-turns must have a stabilizing hydrogen bond, while this is the case for only a slight majority [17,47]. It is commonly accepted to have less than 7.0 Å (or 7.5 Å) between Cα*_i_* and Cα*_i+3_*; the recent questions of a relaxed distance, i.e., 10 Å [74], suggested that turns are too distorted and did not represent compacted local conformations. Moreover, most of the Cα*_i_*–Cα*_i+3_* distances are observed around 5–6 Å. The information (provided by PROMOTIF; see column 17 in Figure 4) on the presence (or not) of a hydrogen bond is surely sufficient, but not the most accessible. 

The last question concerns the types. After J. Thornton’s final propositions, we had nine types (I, I’, II, II’, IV, VI_a1_, VI_a2_, VI_b_ and VIII) [25,50], with strange numbers and some that have been discarded (III, III’, V and VII) [18]. With the addition of the new types IV, we arrived at 13 types (I, I’, II, II’, IV_1_, IV_2_, IV_3_, IV_4_, IV_misc_, VI_a1_, VI_a2_, VI_b_ and VIII) [19]. Koch and Klebe proposed 41 different β-turns [74] and SCOT around 40 [34,77]; the number is sure to be high for simple usage. As for the potential 1296 combinations of Zhang and co-workers, 583 different types were observed [80]. In an adjacent field, the structural alphabets, the Protein Blocks had an excellent impact because of their simplicity and the quite usable number of structural letters (i.e., 16) [83,84]. Hence, the option provided by R. Dunbrack’s BetaTurn-Tool18, with only 18 β-turns, is a good alternative, minus the difficulty of using it [78]. 

To conclude, what is missing is (i) a good SSAM tool and (ii) a proper 3D visualization. The best option could be an extension to (or an integration into) DSSP [21] to provide a simple method for the researcher to access this information. DSSP has evolved and the last version now includes PolyProline II helix assignment [85], while PROMOTIF is now more than 25 years old [22]. Concerning the visualization, the problems are dual, the main one being to manage overlaps between β-turns and repetitive structures. It would therefore be necessary to have (a) integration in the most classic software, such as PyMOL or Chimera, of the assignment rules (or of reading the outputs of the extended DSSP), and (b) a choice of color for the residues that are exclusively β-turns and another for the overlaps with α-helices and β-sheets. Having this option will make it easier to see the importance of β-turns in proteins. 

It is obvious that β-turns still have a future. They are directly involved in a large number of protein functions [86] and are dynamical local conformations [47]. It is therefore particularly unfortunate that they are so often overlooked in analyses when even the proteins involved in SARS-CoV-2 are full of β-turns [87]. Moreover, as new SSAMs are still proposed [40,88,89,90,91,92,93], studies on this local conformation continue, both within proteins and peptides; the latter have an extremely high β-turn propensity [94,95,96]. A fine and recent example is the famous AlphaFold 2 prediction methodology [97]. Analyses of the 3D predicted human proteome (thanks to the EBI database [98]) showed (*submitted article*) that (i) the percentages of β-turns are found as expected in the protein structural models, (ii) even the different types of β-turns are found with the correct occurrence and (iii) also with good confidence scores (pLDDT), underlining that they are still useful. 

## Data Availability

Not applicable.

## Supplementary Materials

The supporting information can be downloaded at: https://www.mdpi.com/article/10.3390/ijms232012314/s1. References [7,23,30,33,48,51,55,60,68,81] are cited in the Supplementary Materials.
