# Supplementary material for: A Perspective on the (Rise and Fall of) Protein β-Turns
Source: Int J Mol Sci. 2022 Oct 14;23(20):12314. doi: 10.3390/ijms232012314 (PMC9604201; doi:10.3390/ijms232012314)
Supplement: Supplementary file 1 [file ijms-23-12314-s001.zip › ijms-1949054-supplementary.pdf]

Title: A perspective on the (rise and fall of) protein β-turns.

Author: Alexandre G. de Brevern

List 1. *Links to songs presented in the manuscript.* Last accessed 2022-09-14.

1. The Rolling Stones - *Paint It Black - Live official* (Official Music Video); https://youtu.be/50u9bxRwBJI [1].
2. Joy Division - *Atmosphere* (Official Music Video); https://youtu.be/1EdUjlawLJM [2].
3. Dead Or Alive - *You Spin Me Round (Like a Record)* (Official Music Video); https://youtu.be/PGNiXGX2nLU [3].
4. U2 - *With Or Without You* (Official Music Video); https://youtu.be/ujNeHIo7oTE [4].
5. Dire Straits - *Money For Nothing* (Official Music Video); https://youtu.be/wTP2RUD_cL0 [5].
6. Zager & Evans - *In the Year 2525 (Exordium & Terminus)* (Provided by RCA/Legacy); https://youtu.be/l3yDLvp9le0 [6].
7. Ringo Starr - *It Don't Come Easy* (Official Music Video); https://youtu.be/UDcZxEpA9XY [7].
8. Joy Division - *She's Lost Control – Live* (Official Music Video); https://youtu.be/Jcjmp0WUOlk [8].
9. Nina Simone - *Feeling Good* (Official Music Video); https://youtu.be/oHRNrgDIJfo [9].
10. The Doors - *The End (Mono, 2017 Remaster)* (Official Music Video); https://youtu.be/NOW7i7aWQoE [10]).

References

1. Jagger, M.; Richards, K. Paint it black. In *Rolling Stones - Aftermath*, 1966.
2. Summer, B.; Hook, P.; Morris, S.; Curtis, I. Atmosphere. In *Joy Division - Atmosphere*, 1980.
3. Burns, P.; Coy, S.; Hussey, W.; Lever, T.; Percy, M. You spin me round (like a record). In *Dead or Alive - Youthquake*, 1984.
4. Bono. With or without you. In *U2 - The Joshua Tree*, 1987.
5. Knopfler, M.; Sting. Money for nothing. In *Dire Straits - Brothers in Arms*, 1985.
6. Evans, R. In the year 2525 (exordium & terminus). In *Zager and Evans - 2525 (Exordium & Terminus)*, 1969.
7. Starr, R. It don't come easy. In *Ringo Starr - Ringo*, 1973.
8. Summer, B.; Hook, P.; Morris, S.; Curtis, I. She's lost control. In *Joy Division - Unknown Pleasures*, 1979.
9. Newley, A.; Bricusse, L. Feeling good. In *Nina Simone - I Put a Spell on You*, 1965.
10. Morrison, J.; Manzarek, R.; Krieger, R.; Densmore, J. The end. In *The Doors - The Doors*, 1967.
